# Emotion Socialization in Teacher-Child Interaction: Teachers’ Responses to Children’s Negative Emotions

**DOI:** 10.3389/fpsyg.2019.01546

**Published:** 2019-08-14

**Authors:** Asta Cekaite, Anna Ekström

**Affiliations:** ^1^Child Studies, Linköping University, Linköping, Sweden; ^2^Department of Clinical and Experimental Medicine (IKE), Linköping University, Linköping, Sweden

**Keywords:** social interaction, child-adult interaction, emotion socialization, norms and values, emotion regulation

## Abstract

The present study examines 1- to 5-year-old children’s emotion socialization in an early childhood educational setting (a preschool) in Sweden. Specifically, it examines social situations where teachers respond to children’s negative emotional expressions and negatively emotionally charged social acts, characterized by anger, irritation, and distress. Data consisted of 14 h of video observations of daily activities, recorded in a public Swedish preschool, located in a suburban middle-class area and include 35 children and 5 preschool teachers. By adopting a sociocultural perspective on children’s development and socialization, the study examines the communicative practices through which the expressions of negative emotions are responded to and the norms and values that are communicated through these practices. The data are analyzed by using multimodal analysis of interaction that provides a tool for detailed analysis of participants’ verbal and embodied actions and sense-making. The analyses show that teachers responded to children’s negatively charged emotional expressions as social acts (that were normatively evaluated), and the adults instructed children how to modify their social conduct (rather than deploying explicit discussions about emotions). The teachers used communicative genres that prioritized general moral principles and implemented the non-negotiability of norms over individual children’s emotional-volitional perspectives and individual preferences. The teachers’ instructive socializing activities were characterized by movement between multiple temporal horizons, i.e., general (emotional) discourse that transcended the here-and-now, and specific instructions targeting the children’s conduct in a current situation. The study discusses how emotion socialization can be related to the institutional characteristics and collective participatory social conditions of early childhood education.

## Introduction

The present study addresses children’s emotion socialization as part of social interactional practices that take place in a preschool, a setting that constitutes a pervasive part of young children’s lives in post-industrial societies. In Sweden, 84% of children between the ages of 1 and 5 years attend early childhood education, and the average time they spend there is 31 h per week (Skolverket, 2013, 2017). Arguably, this means that emotional experiences children face as part of institutional activities significantly contribute to children’s emotional development (Denham et al., 2012). While emotions are associated with psychological states (individual experiences of happiness, sadness, or anger), and bodily reactions (such as heartbeat frequency or muscle tension), emotional displays or emotional stances, defined as embodied and verbal (evaluative) displays of emotion toward a particular focus of concern (usually somebody else’s action) (Goodwin et al., 2012), are also an integral part of social life with clear normative elements. Human affective engagement is a normative practice, both in the way emotion displays are a learnt and acquired competence, and in relation to cultural normative values they mediate (Harré and Gillet, 1994; Baerveldt and Voestermans, 2005; Demuth, 2013). For children, the normative aspects of affective engagement are essential to their emotional competences and are addressed through socialization into a shared culture of emotions. Emotion socialization is defined as a dynamic process, involving a broad range of social – verbal and embodied – practices, through which caregivers mediate community-relevant ways of expressing and interpreting emotions. It is suggested that young children’s “development of emotion regulation is one of the central goals of early socialization because of its importance to social competence, academic achievement” (Thompson, 2015, p. 173) and psychological well-being. However, while we know a great deal about socialization by parents (and especially, mothers, Denham et al., 2012; Wainryb and Recchia, 2014), there is a certain gap in our understanding of what characterizes children’s emotion socialization in early childhood educational settings and, specifically, how early childhood teachers can act as socializers of young children’s emotional expressions and normatively appropriate conduct.

The aim of the present study is to examine and describe children’s emotion socialization in an early childhood educational setting: a preschool in Sweden. Drawing on video recordings of daily activities, we analyze situations where teachers respond to 1- to 5-year-old children’s negative emotions such as anger, irritation and distress (and regulate children’s emotionally valorized acts). The research questions are: (1) how do teachers respond to children’s negative emotional expressions; (2) how are the social meaning and normative evaluation of such emotional expressions (as social acts) achieved through the communicative practices of teachers and children; (3) what characterizes the interpretative frameworks (e.g., general normative or individual volitional, as well as present or hypothetical, future-oriented) that the teachers deploy in their responses to children’s negative emotional displays.

The aim of the present study is thus not to track the developmental aspects of children’s emotion expressions. Rather, we explore the normative evaluation (and implicit regulation) of children’s negative emotion expressions and related social acts as it is accomplished through embodied practices of social interaction in an institutional setting. In that displays of negative emotions have a propensity to indicate a difficulty or a problem, for example, that “one lacks what one desires or one’s well-being is threatened” (Demuth, 2013, p. 18) or that a conflict arises, they are clearly normatively significant and frequently invoke caregivers’ response. In such cases, the situational and cultural appropriateness of children’s emotionally charged social acts becomes a matter of caregivers’ normative evaluation (Kvist, 2018; Cekaite, in press a). We will discuss how emotion socialization can be related to the institutional characteristics and collective participatory conditions of early childhood education. We use a social interactional perspective (Goodwin, 2018) in order to further the understanding of norms and values related to the expression of emotions promoted in these interactions, as well as the interactional – discursive and embodied – practices used to communicate these norms and values. An additional aim of the study is to demonstrate and discuss how multimodal interaction analysis, that attends to situated character of human conduct and to the embodied features of socialization, can provide a fruitful addition to studies on emotion socialization.

## Theoretical Perspective

Recently, various perspectives (including cultural psychology) have demonstrated increasing acknowledgement that children’s emotion socialization and development are anchored within caregiver-child interactions, and are thereby influenced by sociocultural processes, norms, and values (Holodynski and Friedlmeier, 2010). Related perspectives exhibit an increasing focus on human agency and dynamic features of socialization (Kuczynski and Knafo, 2013). Development of shared values, as well as emotional expressions, is thus conducted through participation in embodied and materially anchored practices of social interaction, more specifically, in particular communicative genres (as recurrent types of social activities, Bakhtin, 1986; Linell, 2009; Demuth, 2013). Moreover, socialization involves not only continuity and conformity (i.e., adherence to and acculturation into common societal norms) but also the possibility of change and the emergence of novelty (Kuczynski and Knafo, 2013, p. 324), and therefore, analytical perspectives can fruitfully pay attention to the interactional emergent features of socializing encounters. This implies that one has to inductively examine both adult instructions and children’s negotiations of norms and values in order to uncover the ways in which adults and children construct and interpret emotional expressions and their normative appropriateness.

Anthropological perspectives on cultural processes of socialization (Ochs and Schieffelin, 1989, 2012; Harkness and Super, 1992) have developed an approach that conceptualizes socialization as a process that involves implicit and explicit socializing actions and encounters. Explicit socialization is associated with instructional actions (accomplished by adults or by children’s peers) that spell out the prevalent norms and educate children’s conduct. Explicit socializing acts can clarify and correct children’s actions and emotional expressions and provide various kinds of instructions about affective and social values. Implicit socialization is associated primarily with ways of acting that constitute a common, non-explicit way of attending to emotionally valorized actions and their appropriateness. While a significant part of this research has been directed at the use of language resources as emotional stances, connecting socialization to language and culture (Ochs and Schieffelin, 2012), recent theorization of human sociality directs attention to human actions as embodied, both corporeal and language based, and located in material space (Goodwin, 2018). Goodwin et al. (2012) suggest that a speaker’s performance of emotional stance is a situated, sequentially positioned act that displays emotion as socially responsive and consequential for future interaction. It involves the use of intonation, gesture, body posture, facial expressions, and talk. Such a holistic, interactional, and multisemiotic view of emotions differs from a view of emotions as something that resides within the individual and is expressed primarily verbally through meta-level lexical glosses (“sad,” “upset,” “angry,” etc.). Rather, emotions are associated with social acts (located in social interaction), and are responsive to something (e.g., a phenomenon or somebody’s actions) and directed at somebody. They are inextricably linked with social moral and normative orders that simultaneously provide the evaluative framework for the assessment of social meaning and appropriateness of the emotion (Harré and Gillet, 1994). In a situated sense, emotional stances are linked to activities and practices (Goodwin et al., 2012) and the way activities unfold becomes a resource for children’s learning of emotional competence, including how one’s actions and emotional expressions fit the normatively expected organization and flow of activity. In this way, repeated participation in social interaction provides a socioculturally anchored template for children’s emotional and social learning.

### Socialization of Negative Emotions in Family Settings

Negative emotions have received extensive attention in research on young children’s emotion socialization, primarily in studies of families, and especially between mother and child. Emotion socialization involves both socialization for discernment of specific emotions, and emotion regulation as a way to develop emotional competences (Thompson, 2015). Emotion regulation, defined as the ability to handle emotions in order to cope in various situations (Denham et al., 2012, p. 2), is considered one of the major foci and achievements in children’s social development. Research has identified parental strategies such as modeling, responding, and instructing, and it suggests that children, through observation of adults’ emotional conduct, can learn which emotions are acceptable and how to express and regulate them. Parents’ responding to and teaching of emotions involve strategies that instruct children through adults’ responses (validating or criticizing), and inform or instruct children about emotions by linking children’s experiences, situations, and verbal labels into “coherent scripts about emotional experience” (Denham et al., 2012, p. 4) and parent-child talk advances children’s emotion understanding (Thompson, 2015).

Cultural psychological studies have approached the socialization of infants’ negative emotions by examining mother-child encounters in various cultural settings (Friedlmeier and Trommsdorff, 2011). They suggest that infants’ negative emotions are socialized differently by parents who orient to children’s autonomy and self-expression (parents validate negative emotions and scaffold self-regulation) and by parents in societies where self-expression weighs less than subservience to common values (and where parents enact emotional restraint with their children). This research perspective has been modified to more clearly account for situational and communicative resources that express various normative orientations to children’s negative emotions. For instance, Demuth (2013), p. 9, in a discursive psychological study has examined how German middle-class and Nso rural mothers used various communicative genres (Linell, 2009) that positioned the child as “a quasi-equal negotiation partner versus positioning the child as having to obey and comply with a hierarchical setting.” Mothers used various discursive practices: those that mitigated possibilities for overt control by providing rationale, reasoning and maneuvering. These strategies worked to secure an alignment of perspectives between mothers and children. Contrastive strategies were used by (rural) mothers who, in response to children’s negative emotional expressions and acts, implemented “overtly directive strategies” that expressed non-negotiability and “nonacceptance of the child’s behavior” (Demuth, 2013, p. 18).

Furthermore, in a study of Swedish middle-class family practices (parent – child interactions), Goodwin and Cekaite (2018) identified a communicative style that was used in response to children’s negative emotions (in their resistance to parental directives). Negotiations, reasoning, and covert parental control were prevalent and children were given extensive opportunities to re-negotiate parental decisions, by, for instance, using pleading stances to get the parent to align with the child’s desires and wishes. Recurrently, children’s (emotional) autonomy was confirmed and valued by parents.

Parent-child talk can also provide a socialization template for advanced and complex moral reasoning (Wainryb and Recchia, 2014), and some of the studies argue strongly that parent-child moral dialogue provides a more conducive environment for children’s active participation and negotiation of moral discourse (compared to institutional educational settings). Examples of actions toward others that consistently invoke moral reasoning and reactions include harm to another, unfairness, and unequal treatment (Sterponi, 2009, 2014). These actions do not stand alone; rather, they are usually intertwined with negative emotional expressions. Moral and emotional features of conduct become inextricably linked in social interaction and socialization (Goodwin and Cekaite, 2018).

### Early Childhood Education as a Context for Emotion and Moral Socialization

In addition to family, which has been a primary focus of emotion socialization research, institutional early childhood settings (especially in Western countries) have become significant contexts for development and socialization for children from an early age. Educational settings teach norms and values as well as related emotional displays (Cekaite, 2012a,b, 2013), and children learn in institutionalized practices that are characterized by communication and shared activities, anchored in traditions, and shared normative expectations and societal values. Early child care and education constitute social settings that differ from families in relation to the goals of the institution, participant constellations, and the intimacy of social relations. The multiple institutional goals in this collective setting include both care and education. Here, individual volition and general perspective on common good come into a close, and at times, controversial perspective. Participation in educational settings requires children’s appropriation of normative expectations (Evaldsson and Melander, 2017) that guide non-conflictual conduct, including embodied aspects of participation and expressing or concealing emotions. While these norms are not the primary socialization and learning goals for early education institutions, they constitute an inherent part of socialization and learning practice, and are often a responsibility of teachers.

Research on children’s emotion socialization (taking an anthropological perspective) examines normative expectations that characterize teacher-child and peer interactions in these early education settings. For instance, Ahn (2010) in her longitudinal ethnographic study of a middle-class preschool in the U.S. shows that teachers expressed and socialized children into emotion narrativity, and taught children to verbalize and label negative emotions (“I’m scared”) as a proper way of acting and preventing conflicts in the peer group. At the same time, children recycled this socializing message creatively by transforming it in arranging their peer relations (e.g., trying to ingratiate or exclude someone), rather than following the adults’ normative expectations. In other cultural contexts, such as a Korean preschool, it is teachers’ feelings and children’s responsibility for not making the teacher sad that were instilled through teachers’ disciplining practices (Ahn, 2016). In a longitudinal ethnographic study from a Japanese preschool, teachers’ immediate responses to children’s crying in peer conflicts characterized negative emotional expressions as social acts that clearly impeded on the social harmony of the group and had to be stopped (Burdelski, 2010).

Several ethnographic (video-observation studies) from various Swedish educational settings for young children (preschool, kindergarten, and primary schools) show that emotion socialization is characterized by teachers’ readiness to comfort, acknowledge, and show empathy and compassion to the crying child (Cekaite and Kvist, 2017) or, in case of children’s conflicts, readiness to engage in routinized discursive negotiation practices that invite children’s narrative telling of events and experiences (Cekaite, 2012a,b, 2013, in press a; Kvist, 2018). The latter discursive practices can be seen as representative of non-assertive but determined communicative genre. In children’s peer groups, such mundane and recurrent social activities as play constitute a significant social template where social relations are negotiated, and where there is considerable space for negative emotion expressions, normative transgressions, and conflicts (Goodwin, 2006; Danby and Theobald, 2012; Karlsson et al., 2017; Björk-Willén, 2018; Kvist, 2018).

These studies point to the importance of examining socialization in early childhood collective institutions with a particular focus on how negative emotion displays are evaluated in relation to individual and collective action preferences within the institutional normative frameworks of interpretation. Understanding emotional underpinnings of activities requires an examination of the details of interaction, taking into account “the practice in children’s everyday institutions and the conditions the society gives children for development” together with an attempt to grasp children’s engagements, motivations, and perspectives (Hedegaard 2009, p. 64).

## Materials and Methods

### Participants and Setting

The study was conducted in a public Swedish preschool for children from 1 to 5 years old, located in a suburban middle-class area in a middle-sized Swedish town. Public preschools constitute the main early childhood settings for educare and are attended by approximately 84% of children in Sweden (Skolverket, 2017). The commission of preschools is to serve parents with childcare when they work and at the same time to educate the children. Their aims and work methods are defined by Swedish National Curriculum. A holistic approach to child development, learning and emotional well-being is foregrounded. Children’s development of understanding the others’ perspectives and solidarity are defined as important goals for teachers.

The participants at the preschool include five female teachers^1^ (four university educated teachers and one child-carer) and 35 children (19 girls and 16 boys). A total of 15 children were between 1 and 3 years old (nine girls and six boys), and 20 children were 3–5 years old (10 girls and 10 boys). The preschool was initially contacted with a formal request to the municipality’s education department and school leadership regarding their interest to participate in the study. School leaders then informed staff about the study and inquired whether they would be interested to participate. The parents of the children were informed about the study and parents’ written consent was obtained. The research team was external to the setting and had no affiliation to the preschool or municipality.

### Data Materials

The data consists of 14 h of video observations conducted during a period of 6 months. The data were collected for the purpose of investigating the recurrent practices of children’s emotional and moral socialization in early childhood education, as part of a larger project on children’s emotion socialization^2^. The recordings were made using the principle of “unmotivated looking,” but with specific attention given to activities were emotions were expressed or verbally discussed. The researcher visited the pre-school during two periods in 2015, spring and autumn, and got to know the children. During the video recordings, the researcher ensured that the children were comfortable with being filmed by engaging in some initial conversation, and she responded if the children initiated contact. Usually, the researcher mainly took an observing position and did not actively participate in the preschool activities. In cases of physical conflicts between children or prolonged signs of distress, the researcher called the responsible institutional representative/teacher.

The recordings were conducted using a handheld camera. The documented activities were part of the regular preschool day and included (1) free-play activity where the children were able to choose with whom and what to play, and when they spent time together in smaller friendship groups (approx. 6.5 h); (2) teacher-led organized activities such as circle time, snack time or lunches, educational activities such as book reading, arts and craft, and singing (approx. 7.5 h). The use of audio-visual recordings allowed to capture both vocal and embodied conduct and constituted an important resource as “video data enable the analyst to consider how the local ecology of objects, artefacts, texts, tools, and technologies feature in and impact on the action and activity under scrutiny” (Heath et al., 2010, p. 7). It was therefore possible to study the activities under scrutiny over and over again. The re-playability of recordings made it possible to understand how various communicative resources work together and to explore the local organization of practical action and reasoning as sequentially achieved.

### Ethical Considerations

The study was subjected to ethical vetting by a regional committee for research ethics^3^. Written and oral information was provided to staff and parents, and a consent form was signed for those adults who wished to participate (for parents, this consent also included their children). When visiting the preschool, the researcher frequently asked the children’s permission for recording, and the researcher was sensitive to signs of discomfort from the children that could be associated with being observed for the study. To avoid for the participants to be recognized, detailed information about the participants is not provided, and the sketches used for illustrative purposes are anonymized.

### Method and Analytical Approach

The data is analyzed by using multimodal analysis of interaction (Mondada, 2016; Goodwin, 2018) that provides a tool for detailed analysis of participants’ verbal and embodied actions and sense-making practices as ways trough which social and cultural order is achieved in naturalistic, face-to-face interactions. This ethnomethodologically inspired approach is concerned with social actors’ actions and collective procedures of social order, described by attending to the social actors’ endogenous, emic perspectives on social practices, rather than individuals’ intentions. Social actors’ situated meaning-making procedures and their continuous engagement in production of social order are characteristically documented through video-recordings of social practices (Cekaite, in press b).

Multimodal analysis of an interaction considers the sequential organization of language practices, the embodied participation frameworks, objects, and fine-tuning of participants’ attention to these objects, public display of affective stances, as well as the broader, sociocultural features of the institutional setting. The point of departure is not an examination of isolated sentences, but sequences of actions where talk is embedded in and shaped by preceding actors’ actions. By examining sequences of participants’ actions, we can therefore document and describe how social actors make visible for each other the meaning of each other’s actions. This practice of *in situ* sense-making on a turn-by-turn basis takes place in an ongoing activity and therefore the social activity context is a necessary analytical level in the analysis of the participants’ social cultural practices, and their social worlds. A point of departure for studies investigating social activities as embodied and situated in a material context is an acknowledgment that participants make use of a variety of semiotic resources including vocal actions, gaze, gestures, mimics, bodily orientation, touch, and manipulation of objects when building actions together. The interplay between vocal contributions, bodily conduct, and the material surrounding has been described using the metaphor of an ecology (Goodwin, 2003, p. 35) indicating the existence of a number of communicative resources evolving when multiple participants build relevant meanings and actions together. As frequently argued, the various resources used for communication ought to be understood as mutually supporting and co-dependent systems working together when conveying meaning, rather than as distinctive, self-containing meaning making systems possible to investigate as separate entities (Goodwin, 1981, 2007; Streeck et al., 2011).

The social interactional approach emphasizes the importance of emotional stances in moment-to-moment emergence of social situations, in that they contribute to aligning participants into the co-operative organization of a common course of action (Goodwin, 2018). A focus on intersubjectivity as an achievement of the participants on sequential basis is central to multimodal analysis of interaction and is studied through the ways participants themselves display their understandings of each other’s actions and the unfolding course of events: it is in the response to an action that the recipients of this action display their understanding of what is going on (Sacks et al., 1974).

### Analytical Procedure

The analysis began with one of the authors repeatedly viewing the video data and logging sequences where a teacher in some way responded to and addressed children’s negative emotional expressions. The categorization of emotional stances and socializing instructional actions was based on previous research (e.g., Goodwin et al., 2012; Demuth, 2013) and was refined during analysis in relation to the verbal and embodied conduct of the participants. In the selection of episodes, any kind of response or address the teachers directed toward the child’s expression of a negative emotion was included, thereby the data extended beyond the adults’ responses that explicitly verbally labeled and discussed emotions.

The episodes were analytically selected according to when they started and ended and how the teachers dealt with children’s negative emotional expressions. The episode started when a child or group of children expressed a negative emotion and, in response to that, the teacher addressed the child or group of children. The episode ended when the teacher re-oriented to another task or changed the conversational topic. A total of 49 episodes where an individual child or a group of children expressed negative emotions – such as frustration, irritation, or distress – and the teacher addressed the child/children were identified. The episodes were related to a variety of events: children’s peer conflicts, their dissatisfaction with the scheduled activities, or the teachers’ ways of conducting them, as well as mundane instrumental actions such as problems tying shoelaces and serving food. The identified episodes were more common in teacher-led activities (36 episodes) than free-play activities (13 episodes).

In the next analytical step, the episodes were analyzed by both authors according to the participants’ emotional stances, the problem the participants oriented toward, and the teachers’ socializing instructional actions. This analysis revealed that teachers mainly directed their socializing comments and actions toward children’s negatively charged actions rather than explicitly orienting to their feelings. Many episodes (39) were rather short and consisted of the child’s negative emotion-expression; teachers’ response; and a resolution of the problematic situation. A total of 10 episodes involved teachers’ extended responses and instructional acts. Such extended episodes are “information-rich” in that from them “one can learn a great deal about issues of central importance to the purpose of the inquiry” (Patton, 2002, p. 273). The article presents detailed analysis of three extended instructional episodes that are particularly information-rich in relation to teachers’ explicit and implicit approaches to children’s negative emotional stances and actions, and ways in which the teachers elaborated and explicated norms and values.

Extended instructional sequences were chosen because they have a potential to reveal and contribute to understanding cultural and moral orders as relevant aspects that are clarified and spelled out for the children to learn. In this way, the teachers’ explications became analytically available to the researcher, and instructional episodes constituted a *perspicuous setting* (Garfinkel, 2002, p. 181 ff) for the study of human sociality and activities. The extended episodes were qualitatively similar to the shorter ones in respect to the teachers’ socializing actions and their focus on the children’s behavior, disciplining and validation of children’s emotion expression, and reorganization of activities to solve problems. The episodes include both teacher-led activities and free play, and illustrate two overarching characteristics: how socializing instructions oscillate between general principles and situated practicalities and how instructional activities are directed toward children’s actions within multiple temporal horizons. Repeated data sessions and discussions within a research group^4^ with extensive experience of analyzing preschool activities contributed to discerning the specific findings constituting the results, and final interpretations were checked with the research group.

### Transcriptions

The transcripts were produced for the analytic work and for presenting analytic results. Data were transcribed verbatim and translated to English. Embodied conduct relevant for the analyses were included in the transcripts both as descriptions within double parenthesis and with the use of anonymized drawings based on frame grabs from video clips^5^. Short silences are marked with number of second within brackets, e.g., (0.5) for a half second silence or a (.) for pauses shorter than 0.2 s.

## Results

Children’s negative emotion displays were usually associated with social actions that constituted parts of peer interactions, gatherings, or play (see also Kvist, 2018). In peer conflict situations, the preschool teachers were faced with a number of complex tasks that required them to attend to and support children’s emotional needs and well-being, orient toward educational goals of the preschool, and sustain a smooth flow of activities on an organizational level. The teachers were engaged in various participation constellations, and had to both attend to children on an individual basis and supervise the child group. These multiple tasks were intimately related and managed simultaneously as part of the same situation by using a range of interactional socializing practices that implicitly or explicitly managed – corrected, criticized, or instructed – the children’s conduct and emotional expressions.

Notably, there were considerable tensions between the individual children’s actions, emotional experiences and volition, and the collective and general norms of conduct and feelings. The socializing messages and cultural norms that were promoted by the preschool teachers toward the children can be characterized as exhibiting a certain amount of social control and subservience toward social and institutional norms (c.f. Demuth, 2013). The non-negotiable character of institutional norms was instantiated by the teachers through the use of mitigated directive strategies that avoided confrontation with the children. Simultaneously, in extended episodes, these communicative genres (Linell, 2009) supported, invited and presented a certain amount of reasoning, explaining, and listening to the child’s individual or collective perspective. The teachers employed communicative genres comprising reasoning and persuasive mode by using questions, directives, and prohibitives to engage or inform the children; requested their narratives and tellings of their perspective; exemplified hypothetical/future situations; and instructed children’s talk and actions. Yet another feature of the teachers’ socializing instructions oriented to and explicated multiple temporal and causal horizons that connected past untoward event, present emotional display, and conflict resolution, as well as socialization to future conduct. The teachers attended to individual cases and generalized norms of conduct, focusing on specific individual cases or generic types of situations.

In what follows, we will use examples from three group activities to illustrate the teachers’ various strategies to address children’s emotion socialization and the mutual co-construction of social norms through preschool interactions. The examples do not represent mutually exclusive categories or practices. Rather, they demonstrate the communicative instantiation of similar educational practices that were identified in the video-observational data.

### Teachers’ Use of Specific Action as Grounds for Providing General Guidelines

In the preschool setting, the specific children’s negative emotional stances (such as upset or whining) were oriented to, criticized, and corrected by the teachers who stated and explicated the normative transgression (exemplifying, for instance, what constituted appropriate or inappropriate emotional response to particular kind of action). Tensions between an individual child’s emotional stances, including corporeal experiences, and general institutional norms of good conduct and feelings were resolved by the teachers who favored the perspective of the collective. The individual child’s behavior and negative emotional expressions were used as grounds for general disciplining designed to be instructive to both the individual and the larger group of children.

**Figure 1:**
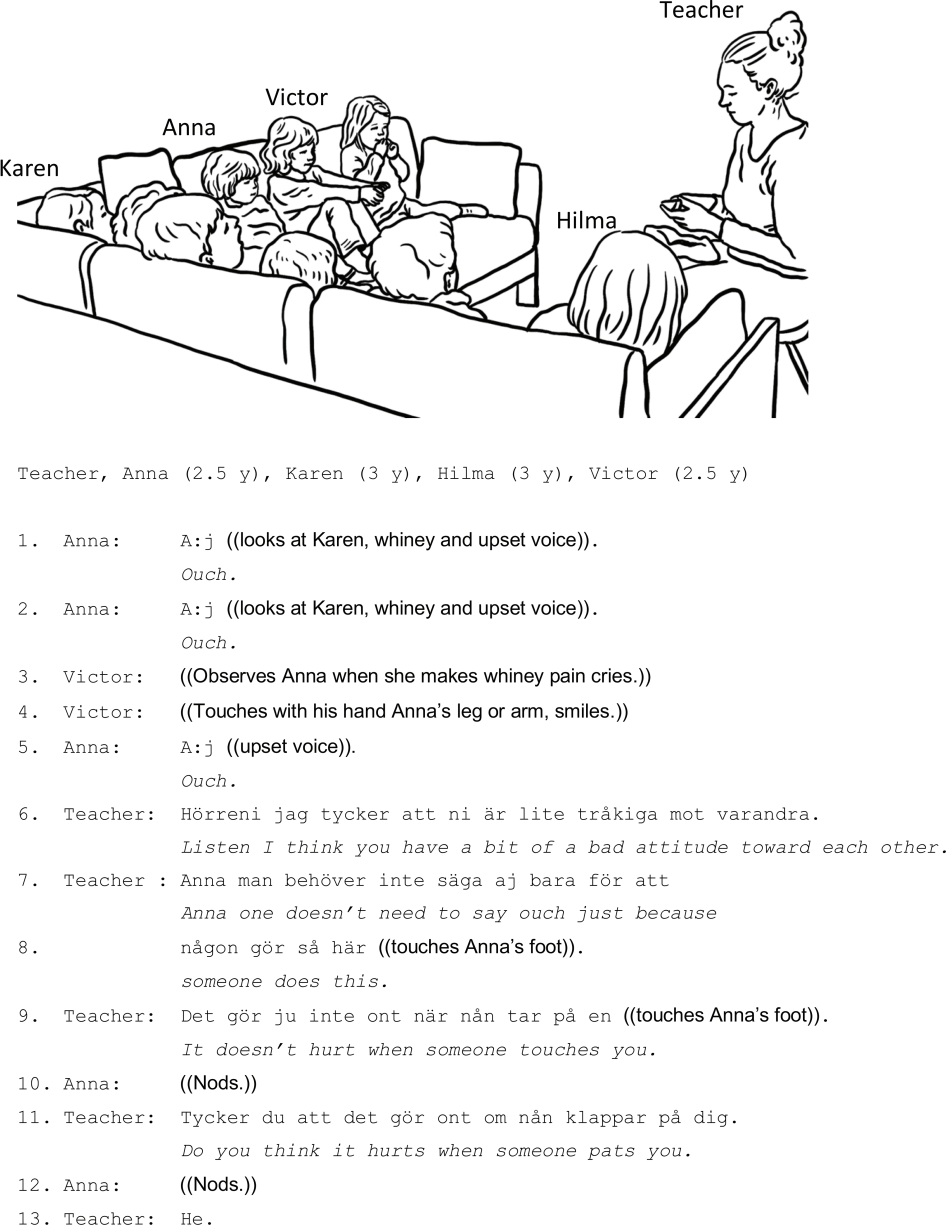
Excerpt 1a.

In Figure 1, nine children wait for the teacher to distribute snacks. As customary, they sit in a sofa closely together, and it frequently happens that they touch each other. Sometimes the corporeal contact provokes the children’s emotionally charged responses. Here, Anna (2.5 years old) sits Karen (3 years old) and Victor (2.5 years old). A bit further away sits Hilma (3 years old), who also participates in the interaction. Several times someone, presumably Karen, engages in physical contact with Anna, who with a whiny, loud voice repeatedly expresses her dislike with “ouch/aj.” When Victor with a cheeky look in his face touches Anna’s arm, she once again complains “ouch/aj.” The teacher then addresses the children as a collective and negatively evaluates their conduct toward each other: “you (plr.) have a bit of a bad attitude toward each other/ni är tråkiga mot varandra.” Upon setting this negative moral evaluative ground, the teacher singles out Anna’s emotional stance and criticizes her negative response to Victor’s touch.

The teacher does not immediately specify which behavior is wrong, with a decisive voice telling the children that they exhibit “bad attitude toward each other/är lite tråkiga mot varandra” (line 06). Rather, the specification of what constitutes “bad attitude” is done by addressing a particular child’s – Anna’s – negative emotional stance and conduct, her whining “ouch” reaction toward Victor’s touch. The teacher states a general norm (“one doesn’t have to say ‘ouch’ just because someone does this/man behöver inte saga aj bara för att nån gör så här,” lines 07–08) as she reaches out to touch Anna’s foot. The teacher’s disciplining (prohibitive that tells what not to do) highlights the discrepancy between the normatively expected tactile sensation of touch and the child’s emotional stance and expression of pain. Notably, the teacher does not invite the explication of the individual child’s perspective on her sensorial experiences, but presents her with a statement as already shared knowledge and non-negotiable common ground (particle “ju” in Swedish indicates the speaker’s assumption that this is shared knowledge): “it doesn’t hurt (ju) when someone touches you/det gör ju inte ont när nån tar på en” (line 09). Notably, the teacher’s instructional disciplining is embodied: she actually re-enacts the way Victor touched Anna, simultaneously subjecting Anna to supposedly similar tactile experience that allegedly caused Anna’s negative affect and whining expression of pain.

However, despite the teacher’s determined stance in her disciplining, Anna disagrees (she indicates that it does hurt, line 10) and the child’s opposing opinion elicits the teacher’s rhetorical question about Anna’s tactile experiences: “do you think it hurts when someone pats you/tycker du att det gör ont om nån klappar på dig” (line 11). Despite the teacher’s focus on Anna’s individual perspectives, she still focuses on more general aspects of touch, and she shifts the verb from a neutral “touching/ta på” to a verb that denotes a soft and intimate touch and that has a clear positive connotation, “patting/klappa.” The intonational pattern of the question suggests that this is a mild challenge to Anna’s stance. The teacher thereby strengthens her stance that Anna’s reaction is not adequate to the action that precipitated it and, despite Anna’s persistence in contrasting opinion (line 12), she terminates their discussion.

As demonstrated, in her instructions, the teacher puts forward a general normative perspective according to which certain negative emotional stances (toward the others’ bodily actions) are inappropriate and have to be modified. The interaction with the specific child aims at socializing and correcting her conduct and emotional expressions on the basis of the general normative expectations, rather than, for instance, a thorough inquiry about the specific child’s subjective corporeal experiences of touch. The general and non-negotiable format of the disciplining statements allows the teacher to provide both Anna, and also the group of children, with concrete examples of an inappropriate emotional stance and conduct, and also locate them within the general normative interpretive framework.

### Children as Co-creators of Moral and Emotional Order

In a Swedish multiparty preschool context, where multiple children – a collective of the peer group – are present (and are potential participants), the teachers were not the sole representatives of the normative interpretation of children’s actions and emotions. The teachers’ socializing instructions, even when they were directed at a specific child, became discursive affordance for the other children who could comment on and join the conversation and address the normative interpretation of specific individual cases. The peer group thereby displayed their abilities to interpret and comment on other children’s emotional expressions and contribute to the development of an emotional order, in such a way necessitating the dynamics of persuasive communicative genre in preschool interactions.

**Figure 2:**
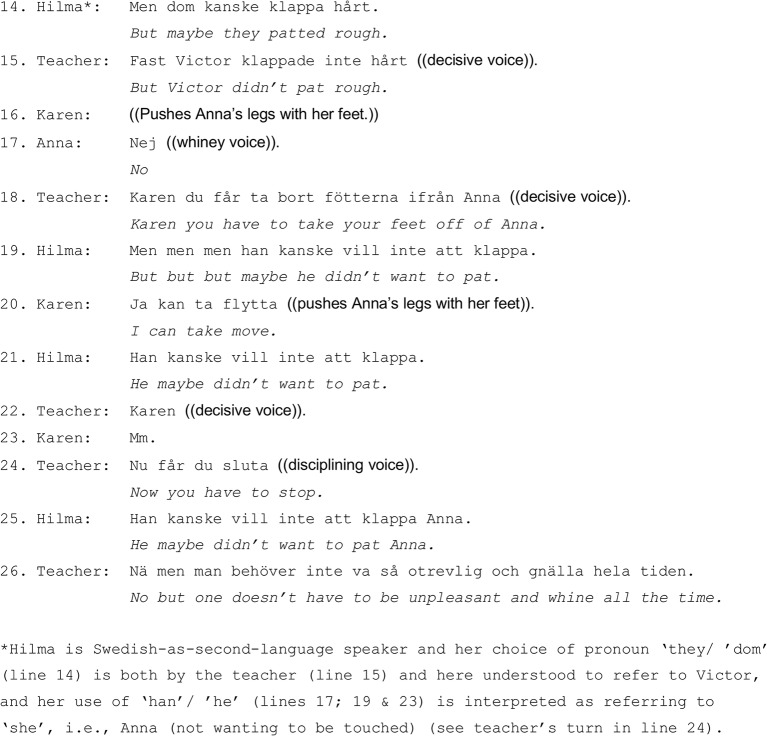
Excerpt 1b.

In Figure 2 (continuation of Figure 1), Hilma re-initiates and sustains the discussion about touch, although the teacher is ready to move on to snacking.

Hilma introduces an alternative view on Anna’s reaction to Victor’s touch: maybe Victor patted Anna in a rough way (line 14). The teacher, however, does not accept Hilma’s interpretation and she instantly states that Victor did not pat Anna in a rough way. In what follows, Hilma pursues the interpretation that foregrounds the individual child’s emotions and volition, several times suggesting that maybe Anna did not want to be patted (lines 19 and 21). She refers to individual preferences rather than general guidelines of how to experience and to respond to a particular kind of touch. However, the teacher is busy disciplining Karen, telling her to stop (lines 18, 22, and 24). Karen puts her feet on Anna’s leg and tries to push Anna away, despite Anna’s loud whining protest. The teacher thus normatively discriminates between various kinds of touch between the children – patting and pushing – and assigns them and their responsive actions, the children’s corporeal experiences and concurrent emotional stances, different values. Pushing (with one’s feet) is considered inappropriate and is decisively disciplined.

When Hilma directs the discussion toward Anna’s preferences and whether or not she wanted to be touched by Victor, this aspect is not subjected to any corrections or instructions from the teacher. Even if Anna did not want Victor to touch her, the teacher takes an accumulative view on Anna’s negative emotional stance, characterizing them as inappropriate, “one doesn’t have to be unpleasant and whine all the time/man behöver inte vara otrevlig och gnälla hela tiden” (line 26). It is the explicit normative orientation toward inappropriateness of continuous whining that concludes this multiparty instructional encounter:

The situation (Figures 1, 2) demonstrates that in a group setting, where many children are present (as agentive embodied subjects with their own emotional and corporeal experiences, perspectives and preferences), it is children’s sensorial and emotional experiences and expressions that become targets for socializing instructions that foreground the collective normative expectations. Unsurprisingly, in the collective of many children, the possibilities to be able to follow individual preferences and express subjective emotional evaluation of the situation (e.g., corporeal experiences) are constrained. During the entire situation, the teacher gives precedence to instruct the specific child, and the group of children on a general level, grounding this socialization project in the specific problematic situation.

### A Co-constructed Framework for Narrative Tellings in Conflicts

The children’s negative emotion displays – annoyance, distress, or sadness – occurred during peer (play) conflicts (c.f. Kvist, 2018) and were recurrently attended to by the teachers who invoked investigatory communicative genres: they engaged the individual children into narrative tellings about their version of the precipitating events. Such tellings presented the children’s individual perspectives and emotional stances toward problematic events, but they were in many cases interactionally steered and orchestrated by the teachers (Cekaite, in press a). The teachers adopted and/or were assigned the moral position to evaluate and to lead the children’s tellings, and then mediate in and resolve the conflict (attend to children’s emotional expressions, instantiate general norms of conduct, while resolving the specific conflict situation as well). The communicative genre of telling multiple perspectives was oriented to by both teachers and children alike, and the children themselves draw on the genres of moral responsibility.

**Figure 3:**
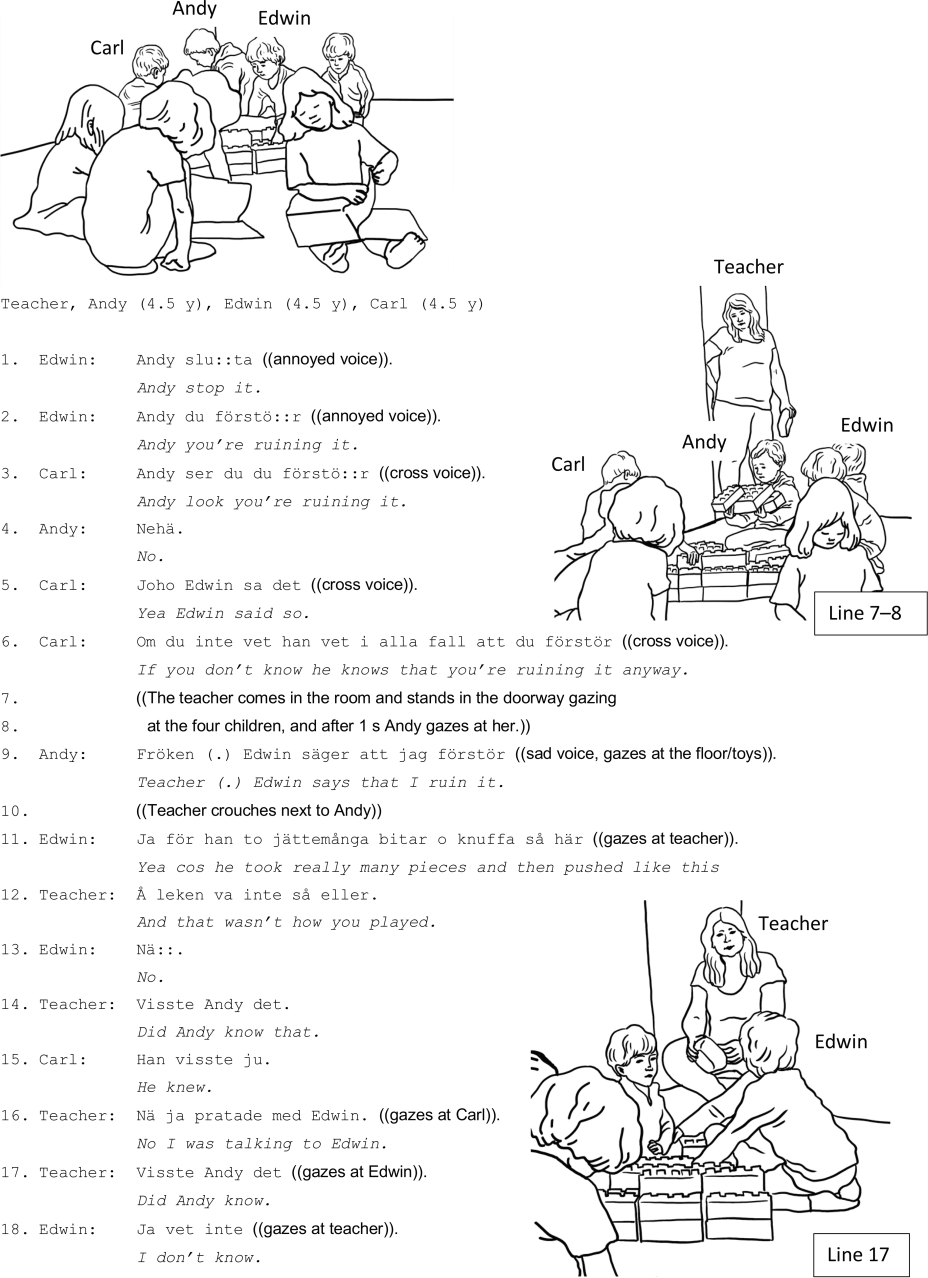
Excerpt 2a.

**Figure 4:**
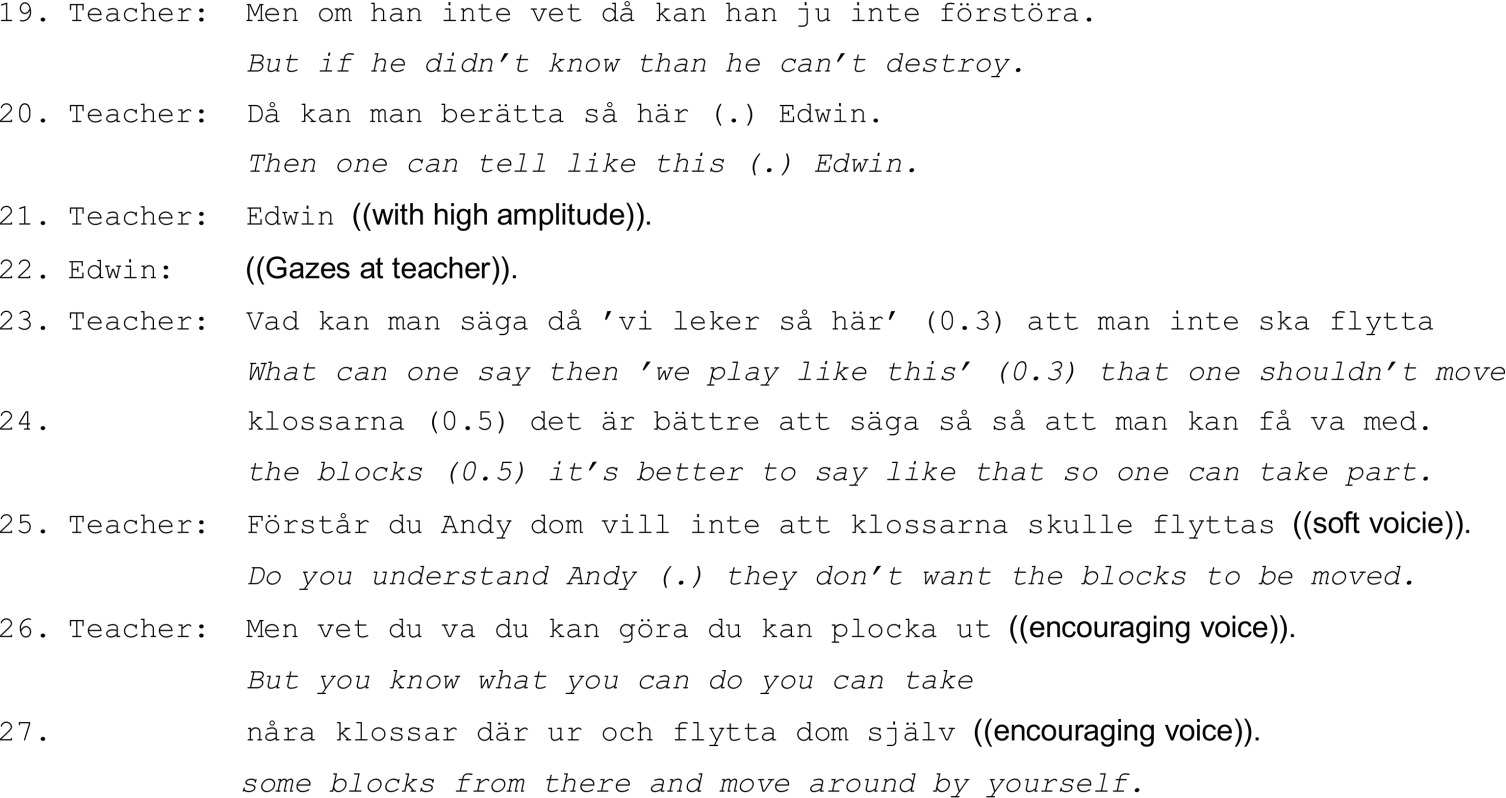
Excerpt 2b.

In Figures 3, 4, three boys (Andy, Edwin, and Carl, 4.5 years old) play with building blocks (the teacher is in an adjacent room). Edwin and Carl, with whiney and annoyed voices, accuse Andy of destroying their play. The children’s conflict – collective accusations and blame denial – continues for some time, and it is colored by several children’s displays of negative affect (lines 1–7).

During the boys’ accusations (lines 01–06), the teacher appears in the doorway (lines 07–08) but she does not address the children. Nevertheless, after just a few seconds, Andy addresses the teacher and reports to her Edwin’s accusation (line 09). He seems to take the teacher’s presence as a request for information or a possibility to report his version of the peer problem. The teacher then crouches next to Andy and now it is the opposing party, Edwin, who articulates his own version of what has happened (line 11). He admits to having accused Andy of destroying the play, but he adds an account that provides a rationale for his accusation: Andy has allegedly taken “really many pieces and pushed/jättemånga bitar å knuffa” (line 11). Andy’s and Edwin’s way of both telling their respective side of the problematic situation to the teacher without the teacher’s prompt demonstrates a routine way of conflict resolution in Swedish preschool; the teacher is positioned as a moral authority responsible for resolving and mediating in children’s conflicts, and the children articulate their version of events.

The children’s own individual versions of events are pursued further by the teacher, who asks Edwin a follow-up question: “and that wasn’t how you played/å leken var inte så eller” (line 12) (she refers to Edwin’s claim that Andy has taken many pieces and pushed). The next step in the investigation concerns the teacher’s leading it toward the issues of responsibility and blame, causally linked to intentionality and prior knowledge. The teacher addresses what becomes the central point of their talk: did Andy know how play was supposed to be (line 14).

As demonstrated, the teacher leads the children’s interactional moves through questions, reasoning, and negotiation, thereby facilitating the children’s tellings in relation to the normative expectations of the classroom, and provides simultaneously positive support. Carl’s statement about Andy’s intentional misconduct is rejected by the teacher who wants to hear Edwin’s version. Upon Edwin’s response that he does not know (line 18), the teacher shifts from investigation and focus on hearing and letting the children narrate their individual perspectives, to a socializing instruction that attends to general and future-oriented features of appropriate conduct.

### Teachers’ Design of Instruction to Address Multiple Temporal Horizons

Following the teacher’s investigation of the children’s conflict situation and their negative stances, various solutions were proposed. Notably, solving current problems in the peer group involved not only orientation toward the specific situation but also the teacher, in a typical socializing instruction, reached out to the future, where similar situations could occur. In this way, the children’s individual perspectives were transformed and re-interpreted within the light of common norms of appropriate conduct that served as a ground for modeling the children’s conduct and language use in the future. A problem in the present temporal horizon was used to provide specific pedagogic guidelines on how children should solve similar problems in hypothetical future situations. Importantly, handling this conflict situation in the present also necessitated the teacher to secure the children’s here-and-now adherence to normatively appropriate actions and their participation in institutional activities.

The teacher instructs the children about how to act in a similar hypothetical play situation: based on the children’s versions, she formulates a conclusive non-negotiable statement and deflects Andy’s blame by explaining the causal link between knowledge, intentionality, and moral responsibility (line 19). The teacher does not explicitly attend to, correct, or affirm, the children’s negative emotional displays (the boys’ irritation and accusations of Andy, or Andy’s distress), although implicitly she does not approve of the reason for their accusations and negative emotional stances. Instead, the teacher instructs the children by invoking a hypothetical situation concerning how the boys can act and talk in a similar situation in the future “what can one say then ‘we play like this’ (0.3) that one shouldn’t move the blocks/vad kan man säga då ‘vi leker sähär’ (0.3) att man inte ska flytta klossarna” (lines 23–24). She enacts what one could or should say by using a generic description “we play *like this*” (line 23) and ties it to the current problematic play: “that one shouldn’t move the blocks/att man inte ska flytta klossarna/” (lines 23–24). The teacher also adds an explanation that puts the proposed line of action into a positive evaluative perspective toward the norm of inclusiveness in the peer group “it’s better to say like that so one can take part/det är bättre så att man kan få va med” (line 24). The teacher uses the style of reasoning that, rather than imposing a hierarchical normative rule, works in a persuasive mode that includes reasons and also highlights positive evaluation and advantages for individuals, or for common good.

The teacher then orients to Andy (who is now sitting turned away from the boys) and uses a softer voice (lines 25–27), explaining the specific actions that have caused the initial play conflict and the boys’ implicated negative emotion: “do you understand Andy they don’t want the blocks to be moved/förstår du Andy dom vill inte att klossarna skulle flyttas” (line 25). Explanatory mode in handling the children’s negative emotional stances and conflict is used in the teacher’s explicit articulation of the causal links between the boys’ actions and conflict, and the children’s volition and individual perspectives are made explicit. Here, an overlap between the multiple prevailing temporal horizons – past, future, and present – becomes apparent as the teacher addresses and explains Andy how he can play now, a solution that does not involve Andy conforming with the rules of the play and joining the other children. As the teacher handles the current problematic situation, one of the tasks for her is to reintroduce the child here-and-now into the institutional activity, and the perspectives of an individual and collective norms are renegotiated (lines 25–27).

### Persuasive Explanatory Talk and Non-negotiable Social Rules

The children’s negative emotional stances (marking their conflictual actions) during peer activities were also managed by the teachers through invoking and articulating institutional non-negotiable norms (for instance, the principle of fairness, inclusiveness, and sharedness of toys and objects) in the case of a specific conflict. Invocation of such norms was usually associated with the teachers’ solution of the conflict that did not leave the children many opportunities to renegotiate the norm according to their own benefit (individual children’s wishes and standpoints regarding the specificities of the conflict). However, the teachers’ general normative orientation did not prevent the young children from pursuing or arguing their individual cases (e.g., their desires) with negatively valorized emotional stances. Responsive to that, the teachers employed a range of persuasive argumentative interactional moves that in various ways spelled out for the disappointed child the institutional rationale and in such ways socialized the children to compliance with the demands of existing social norms, as well as societal and institutional ideologies.

**Figure 5:**
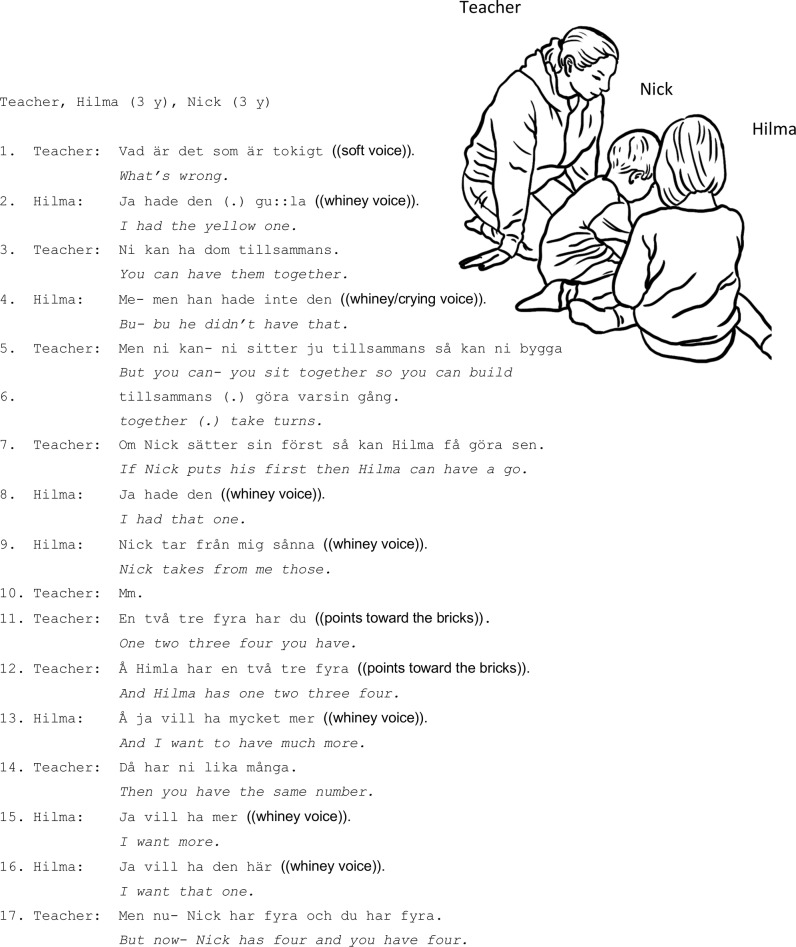
Excerpt 3a.

**Figure 6:**
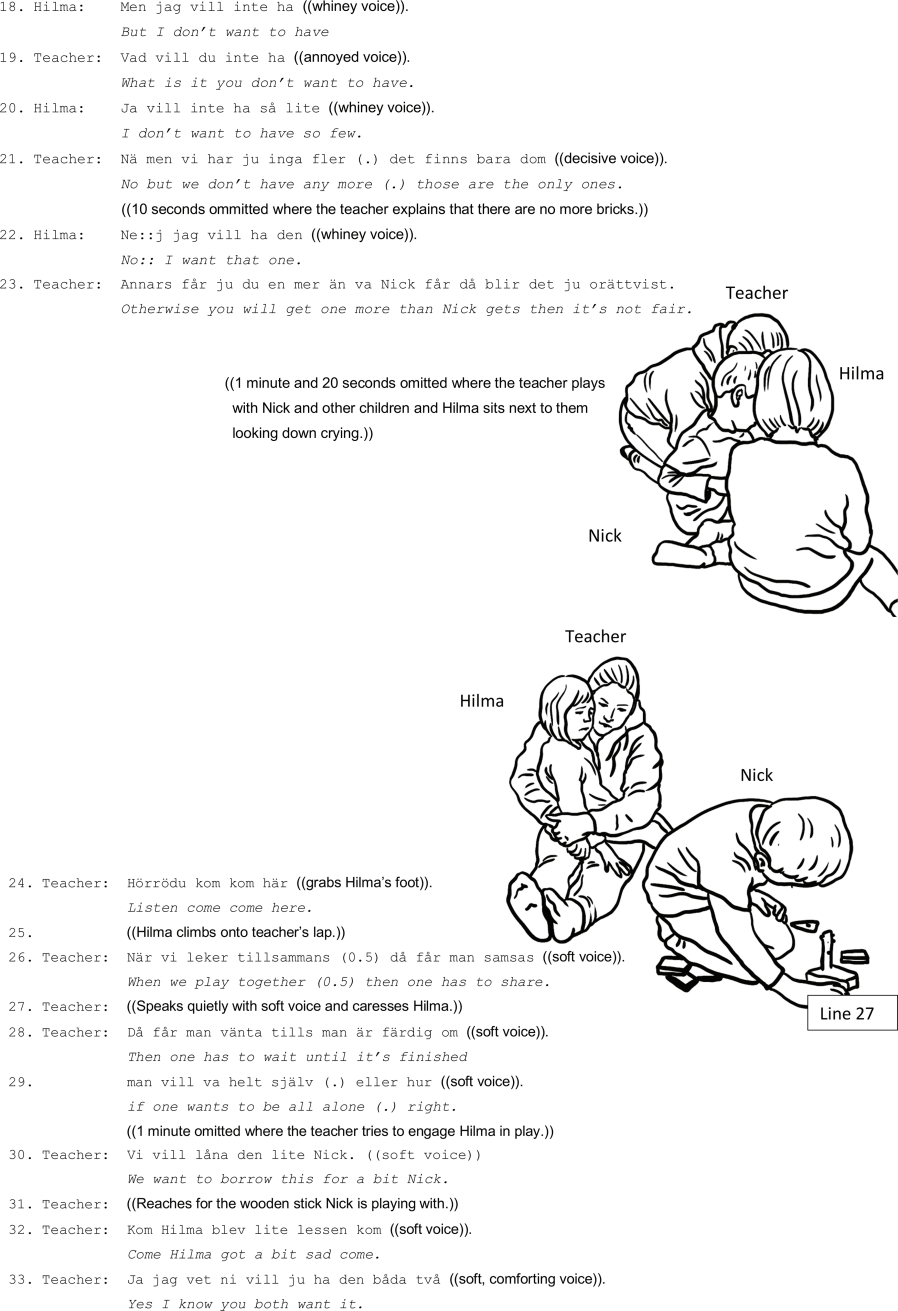
Excerpt 3b.

In Figures 5, 6, Hilma (3 years old) and Nick (3 years old) play with wooden bricks and start arguing about their possessions. Hilma has several times complained about the distribution of bricks with a whiney and annoyed voice. The teacher intervenes in the conflict by asking Hilma and Nick what is wrong (soliciting the children’s individual perspectives, c.f. Figure 3). However, she soon moves on to solve their problem. The problem resolution outlines potential and preferable course of action for the children on the basis of the general institutional norm of fairness, i.e., that toys should be shared or distributed equally.

The teacher does not proceed with the investigation about what has happened (c.f. Figure 3). Instead, she provides a suggestion for how to solve the problem (implicitly referring to the preschool norm of inclusiveness and fairness): Nick and Hilma should play with the bricks together (line 03). Hilma, however, with a whiney voice, continues to complain, and accuses Nick of taking her bricks (lines 04, 08, and 09). These complaints are not investigated or attended to by the teacher who persistently suggests a general solution – children should and can play together (lines 05–07). As mentioned earlier, the teacher presents a number of reiterative suggestions of how to play in ways that adhere to the same norm of sharing and inclusiveness in the preschool: the teacher shifts between suggesting that Nick and Hilma should play together and demonstrating the equal number of bricks that are available to each child. For instance, the teacher exemplifies the norm by counting the bricks aloud (Nick and Hilma have the same number of bricks, that is considered to be the fair way of dividing toys). Hilma, however, does not comply and with a whiney tone of voice she repeatedly states that she wants to have more than four bricks (lines 13, 15, and 16). Instead of addressing Hilma’s viewpoint or her negative emotional stance, the teacher retains the norm of sharing fairly (lines 11, 12, 14, and 17). In doing so, she implicitly rejects Hilma’s individual emotional-volition perspective: the children’s individual desires and wishes have to be modified in the light of the institutional moral order of social solidarity.

### Moral Implications of Children’s Negative Emotional Expressions: Differences in Whiny and Upset Emotional Stances

In preschool teacher-child interactions, the children’s negative emotional stances were interpreted and evaluated as relevant, morally appropriate or not, with a focus on the type of emotion and their action referent in the particular social situation. Whereas the institutional norms were usually an important non-negotiable guideline for social control according to which children’s individual desires were implicitly or explicitly socialized by the teachers in order to foster the subservience to the normative expectations of the community, the child’s individual emotion-volition acts could gain weight, albeit not easily. This could happen in situations when the children displayed continuous upset and excluded themselves from the participation in the preschool activities. Such disharmony disturbed the balance between the smooth flow of preschool activities and the children’s satisfaction, invoking the teachers’ responsibility to support of the child’s emotional well-being and sustain emotional relatedness between the teacher and the child. Institutional norms were thus intimately linked to the type of children’s emotional expressions and the teachers’ institutional responsibilities. There were various sets of norms related to children’s expressions of sadness and distress, compared to stances displaying whininess and irritation.

In Figure 6 (continuation of Figure 5), Hilma keeps complaining in a whiney tone of voice. This time she explicitly refers to her individual desires (“but I don’t want to have/men jag vill inte ha,” line 18). The teacher addresses Hilma’s complaints and for the first time enquires about her individual perspective.

When Hilma whiningly claims that she does not want to have so few (bricks) (line 20), the teacher, with a decisive, disciplining voice, explains that there are no more bricks available (yet again, she does not give in to Hilma’s individual desires that digress from the institutional norms of sharing). About 10 s of similar interactional moves follow (omitted in transcript). Through her voice, the teacher clearly indicates her irritated stance toward the child’s persistent claims of individual desires, and she makes an explanatory blame ascription “otherwise you will get one more than Nick gets then it’s not fair/annars får ju du en mer än va Nick får då blir det ju orättvist” (line 23) that explicates the normative fallacy in transgressing the norm of fairness. At this point, the teacher assertively terminates her discussion with Hilma and she starts playing with Nick and other children. Hilma, however, does not re-engage in play: she sits alone, and her sad facial expression and bodily posture signal that she is upset and she is silently crying (1 min and 20 s omitted).

As the child in distress excludes herself from social interaction, the teacher re-establishes her conversation with Hilma by using a comforting, softer tone, and by inviting Hilma to sit on her lap (lines 24–25). The teacher uses common soothing practices and touch embrace (Cekaite and Kvist, 2017) as a corporeal hub of intimacy and compassion. Notably, while she validates the child’s negative emotional expression through intimate comforting acts (by embracing her and putting her cheek next to Hilma’s cheek while drying her tears), she still sustains the normative orientation of the educational institution: in a very soft voice she explains the rules of the preschool where the children need to take turns and share the toys when they play together (lines 30–33). The way the teacher responds to Hilma’s claims of individual desire and her concurrent emotional stances (c.f. Hilma’s whiny stance in 18, 20, and 22; Figure 5) differs primarily in the affective valorization of the teacher’s talk. This time she renders a detailed and long explanation of how the children have to act if they play together: “then one has to shared/då får man samsas” (line 26). Unsurprisingly, playing together requires downgrading one’s own desires and perspectives and even if children wish to play on their own, one has to curb and restrain one’s desires, at least temporarily (lines 28–29).

However, the problem – Hilma’s desire to have more bricks and her negative emotional stance of upset – has not changed and finally, the teacher suspends the norm of sharing: she asks Nick to lend Hilma a toy he is using. Notably, such digression from the institutional norm is not easy and requires the teacher to interactionally engage in moral relational work toward the group of children: the teacher’s reason-giving involves her appeal to Nick’s empathy because “Hilma got a bit sad/Hilma blev lite ledsen” rather than a principle of fairness (line 32). As demonstrated, the child’s shift in emotional stance – from whiney and annoyed to extended upset and self-exclusion – seems to invoke a new moral order that changes the course of the play activity and gives the child access to the desired object *via* the teacher. Institutional norms and institutional morality (collective vs. individual) are susceptible to attend to the emotional states of the children – whiney or upset – but are not suspended easily.

## Discussion

The present study has examined how teachers in a Swedish preschool responded to children’s negative emotional stances. By engaging in detailed interaction analyses (Goodwin, 2018), we have explored the practices in which emotion and moral norms were co-constructed through embodied social interaction between teachers and children. We have conceptualized these processes as communicatively realized socialization (and regulation) of children’s negative emotions. As demonstrated, in teacher-child encounters, children’s negative emotional stances (embodied and verbal social acts) were evaluated in terms of relevance and normative appropriateness. Based on the type of emotions expressed, a particular communicative genre was deployed to resolve the social situation. Notably, the study did not aim to document the developmental outcomes. Rather, by taking into account the social-ecological conditions of the preschool as a collective institutional setting with multiple – educational and social – goals, we have examined and highlighted the typical variety of teachers’ socializing instructional responses toward children’s actions that were colored by their negative emotional stances.

As demonstrated, the teachers’ socializing instructional work was conducted in different ways that were intertwined with a number of social-relational and institutional concerns. The teachers used both explicit and implicit socializing strategies (Ochs and Schieffelin, 2012). Implicitly, they modeled and responded with their own emotional stances toward the children (Denham et al., 2012), and through disciplining or comforting stances, rejected (Figures 1, 5) or validated (Figures 4, 6) the children’s negative stances and actions. Explicit discursive instructions about emotions *per se* (so called “emotion narrativity,” language labels identifying specific emotions, see Ahn, 2010 on an American middle-class preschool; Thompson, 2015, on parental conversations about negative emotions as strategies for emotion regulation) were not notably present in the data. Rather, explicit socialization strategies dealt with the normative aspects of the children’s conduct, and the explication of social rules. Here, it is notable that emotional stances were inextricably linked to social actions, and the teachers’ normative evaluation immersed the children into the lived experiences that acting and feeling were intertwined within concrete courses of actions. Teacher responses to children’s emotion-linked actions, rather than explicit emotion instructions and the use of emotion labels, characterized the preschool setting.

### Communicative Genres of Preschool Emotion Socialization

#### General and Individual Perspectives

Our study shows that preschool, as a collective institutional environment, presents a specific social environment with its own characteristic communicative genres of children’s emotional and moral accountability. More specifically, preschool teachers recurrently used a communicative genre where general moral and emotional principles were prioritized over detailed explications of individual children’s emotional-volitional perspectives and specific conduct. A prevailing characteristic of the preschool teachers’ instructive socializing activities was the continuous shift between general pedagogic (emotional) discourse (that transcended the current situation and was at times formulated as hypothetical situations, see Evaldsson and Melander, 2017), and specific instructions targeting the children’s conduct and (emotional) experiences in a current situation. Specific emotions and current conflict situations were used as points of departure and opportunities to engage in wide-ranging instructions that extended beyond the current situation. In other words, an individual child’s emotional experience or conduct was usually not investigated in any detail, but used as a starting point to articulate social norms of the preschool. They were incorporated as examples in communicative projects that aimed to be instructive to the larger group of children (Figures 1, 3, 4). For instance, the recurrent communicative genres of investigating and hearing multiple individual perspectives in conflict situations, where children were encouraged to articulate their own version (and were exposed to different perspectives) on a problematic event (Figures 3 –5) allowed the teachers to avoid conflict and refrain from open social control (e.g., Cekaite, 2013, in press a; Kvist, 2018, on similar practices documented in other types of educational settings). At the same time, the children’s tellings were guided by the institutional perspective through the teachers’ leading questions (see contrasting studies by Burdelski, 2010; Ahn, 2016).

#### Non-negotiability of Norms and Teachers’ Persuasive Explanatory Strategies

The present study, conducted in a Swedish preschool, shows that the teachers used different communicative genres, compared to Swedish family parent-child interactions (Demuth, 2013; Goodwin and Cekaite, 2018 on mothers’ responses to infants’ negative emotions). In families, negotiations and covert parental control were present, and parents confirmed and validated children’s (emotional) autonomy. A communicative style that draws on non-negotiability of norms (e.g., general rules of the preschool related to fairness, inclusiveness, non-negotiability of “property” rights) was prevalent. It gave minimal opportunities for the children’s success in renegotiation and also for emphasis on their individual preferences, desires and emotional stances (Figure 5). It is notable that compliance with the norms of the preschool was expected and that numerous persuasive explanatory strategies were used in order to achieve the child’s compliance (Figures 5, 6). The teachers took on the responsibility to interpret and assign the children’s negative emotion stances a normative value, and thereby to confirm or reject their relevance (Figures 1, 4–6). This was done either by disciplining or, in contrast, validating children’s actions and emotion displays. Notably, while the ways people experience and feel in a specific situation are often considered to be subjective and something that varies between individuals, the teachers were able to take a position as an authority who could evaluate, confirm or disregard the children’s individual experiences (Figures 1, 2). Institutional norms were usually presented as non-negotiable guideline for social actions and they simultaneously served as interactionally situated guidelines according to which children’s individual wishes and aspirations were socialized by the teachers (see also Kvist, 2018 on teachers’ similar responses to children’s crying). Thus, life in a preschool valued certain conformity to general norms (in contrast to extensive possibilities for renegotiations documented in Swedish family interactions, Goodwin and Cekaite, 2018).

The children’s individual emotion-volition acts were sometimes taken into account by the teachers in their resolution of the problematic situations in the children’s peer group. This happened primarily in situations where a child displayed a continuous emotional stance of upset. There were thus specific set of norms related to children’s expressions of sadness and distress, compared to their whiney or irritated stances. Institutional norms and moral frameworks were, in this sense, intimately linked to the type of emotional stances taken by children. Moreover, as preschool activities were routinely organized as multiparty interactions (including groups of children), preschool teachers were not the sole interpreters of children’s emotional conduct. Peers routinely commented on and evaluated their peers’ emotional expressions and conduct, and in this sense, contributed to the development of emotional discourse and moral order in the preschool.

#### Multiple Temporal Horizons

While preschool teachers gave precedence to general guidelines that were designed to be applicable in future situations, the necessity to deal with a conflict situation in the present imposed a requirement to assure that the children acted and participated appropriately in the institutional activities in the present, i.e., here-and-now. In this way, multiple prevailing temporal horizons – past, future, and present – became inherently intertwined in preschool teachers’ instructional socializing actions. They show how children’s emotion and moral socialization extends into the abilities to view oneself as a social persona in a temporally multi-layered, i.e., multi-scalar perspective. The teachers immersed the children into interactional practices that furthered their understanding of causal and temporal links between what the participants’ (teachers or children) deemed as appropriate or inappropriate actions and emotional stances. Here, a division between general normative guidelines and specific, here-and-now, individual resolution of a problematic situation became apparent and pertinent, and the children could be experiencing somewhat divergent and ambiguous socializing messages (Figures 4, 6). General norms for appropriate conduct were at times disregarded in the service of a satisfactory resolution of the current (emotional) problem.

### Limitations and Advantages of the Present Study: Emotion Socialization From Multimodal Interaction Analysis Perspective

The findings of the study can be seen in the light of some limitations, mainly related to sample size and data material, the short term of data collection as well as the use of time-consuming inductive analytical method. The video-ethnographic data does not allow the study to be regarded as a full-scale ethnography that can provide a rich account of participants’ motives and normative world views. Also, the data are based on video observations from one regular Swedish preschool and therefore it does not provide grounds for representative generalizations about the normative specificities of Swedish early childhood education as such, but discussion of results shows significant similarities to emotion socialization practices documented in other studies from Swedish educational institutions (see Cekaite, 2012b, 2013, in press a; Evaldsson and Melander, 2017; Björk-Willén, 2018; Kvist, 2018). In that the data collection did not involve a longitudinal design, we are not able to document and discuss the (factual) outcomes of the socializing instructional practices and have limited possibilities to causally link certain practices to specific learning outcomes and children’s development emotional competences and emotion regulation. Moreover, the detailed interactional analytical perspective relies on inductively emergent categories and does not strive after statistically representative results, or what is commonly considered as replicable study design related to hypothesis testing.

However, viewed from a methodological perspective, the current study provides a novel insight into how multimodal interaction analysis can be used to explore communicative practices and can add to the understanding of traditional psychological topics. The social interactional approach adopted in this study focuses on social and psychological phenomena by attending to and analyzing so called emic, participants’ perspectives. The use of multimodal interaction analysis highlights that emotion socialization is multifaceted: it clearly reaches beyond language use and verbal emotion labels, and is largely orchestrated through multiple semiotic means (thereby extending beyond discursive emotional labels). Some of the advantages of the present study involve the use of the particular analytical method. Examining interactions between teachers and children from the perspective of a multimodal interaction analysis has emphasized the embodied and contextual character of emotion socialization and rendered socialization as temporally unfolding multisemiotic interactive actions. Through the close examination of embodied actions of the participants, the study revealed that explicit talk about emotions and emotion scripts did not dominate the present early education setting (in contrast to the studies suggesting that verbal practices in caregiver-child conversations about emotions and moral issues enhance children’s emotion regulation and moral development, see Wainryb and Recchia, 2014; Thompson, 2015). While the absence of explicit emotion talk can be seen as characteristic to the particular preschool, the current findings that emotions are primarily interpreted in terms of the appropriateness of children’s social actions can suggest a relevant avenue for further exploration of how emotion socialization are conducted in embodied, multisemiotic, and social interactions.

Analysis of video data from a multimodal interaction analytical perspective, deployed in the present study, allowed us to examine how children and teachers display emotional stances by using a variety of resources, including – apart from talk – prosody, gestures, gaze, facial expressions, bodily posture, haptic formations, and spatial positioning. Emotion socialization in preschool activities is inherently multisemiotic: it targets emotion displays as embodied and situated. Analytical focus on the unfolding of emotional stances and the sequential organization of the participants’ interaction, which is at the heart of multimodal interaction analysis, has highlighted and uncovered the indexical link between emotional stances and social activities. Moreover, the method used in the presented study demonstrates that emotion socialization in preschool does not only target children’s conduct and their emotional expressions, but also to some extent involves children’s embodied experiences – a perceptual socialization of sensorial competence (e.g., Figures 1, 2). The present study argues that the ways in which embodied, spatially and materially embedded social activities unfold serve as cultural resources and interactional templates for children’s emotional and normative development. Detailed multimodal interactional analysis provided possibilities to discover how children were introduced into normative frameworks of sensorial understandings and were taught how to interpret their embodied sensations. We suggest that a broadened perspective, including embodied and spatial dimensions of social actions as both resources and targets for emotion socialization, as has been demonstrated here, could deepen our understanding of how a shared emotional world is constituted (Goodwin, 2018).

## Ethics Statement

Regionala etikprövningsnämnden i Linköping, Avdelning för prövning av övrig forskning. (“Regional ethical board in Linköping, Section for probation of general research”) Affiliation/Address: Linköping University Hälsouniversitets kansli Sandbäcksgatan 7 581 83 Linköping, Sweden.

## Author Contributions

AC and AE contributed to the design of the study, the analysis of the results, and the writing of the manuscript. Authors are listed alphabetically.

### Conflict of Interest Statement

The authors declare that the research was conducted in the absence of any commercial or financial relationships that could be construed as a potential conflict of interest.

## Footnotes

^1^In this article, the term teacher is used to refer to all educators working at the preschool.

^2^The data was collected by Disa Bergnehr, a researcher in the project “Communicating emotions, embodying morality,” financed by the Swedish Research Council (PI Asta Cekaite).

^3^Regionala etikprövningsnämnden i Linköping, Avdelning för prövning av. övrig forskning. (Regional ethical board in Linköping, Section for probation of general research). Affiliation/Address: Linköping University Hälsouniversitets kansli Sandbäcksgatan 7 581 83 Linköping, Sweden.

^4^The research group consists of researchers and doctoral students at the Department of Thematic Studies – Child Studies, Linköping University.

^5^The figures (line drawings) are original and have not been used, published, or reproduced from before. They are produced for our research study specifically.
